# Visual ethnographic documentation: a novel tool for mycetoma awareness and advocacy

**DOI:** 10.1093/trstmh/trac048

**Published:** 2022-05-30

**Authors:** Ahmed Hassan Fahal, Linet Atieno Otieno, Eiman Siddig Ahmed, Taysir Kashif, Mahmoud Khalid, Ahmed Hussein Mahmoud, Lameck Ododo, Thomas Kyalo, Sahar Mubarak Bakhiet

**Affiliations:** Mycetoma Research Center, Soba University Hospital, University of Khartoum, Sudan; Drugs for Neglected Diseases initiative, Nairobi, Kenya; Mycetoma Research Center, Soba University Hospital, University of Khartoum, Sudan; Mycetoma Research Center, Soba University Hospital, University of Khartoum, Sudan; Mycetoma Research Center, Soba University Hospital, University of Khartoum, Sudan; Mycetoma Research Center, Soba University Hospital, University of Khartoum, Sudan; Lameck Ododo Photography, Nairobi, Kenya; Uzi Video, Nairobi, Kenya; Mycetoma Research Center, Soba University Hospital, University of Khartoum, Sudan

Mycetoma is an extremely neglected tropical disease that still receives insufficient attention from international medical and health communities, non-governmental organizations and research funders. It has serious health and socio-economic impacts on patients, families, communities and the health system in endemic areas.^1–3^ In this communication, we highlight the stories of two mycetoma patients, El Safi and Albagir, through visual ethnographic documentaries, recording their sufferings and the impact of the disease on their families and how this approach can be used in the future as a novel tool for mycetoma advocacy.

The first patient is a singer and the second has both physical and mental disabilities. Both had a massive long-standing and neglected foot mycetoma that resulted in below-the-knee amputation. The indication for amputation was multifactorial, including the massive lesions at presentation. Factors contributing to late presentation include low socio-economic and health education status, remote locations, lack of access to treatment and the effects of the coronavirus disease 2019 (COVID-19) pandemic.

## Study team

The study team consisted of a visual anthropologist, film director and editor, camera operator, two medical doctors and several healthcare providers. After obtaining written consent, the patients and their families were carefully interviewed and closely observed during their daily activities at home and in their village, at the Mycetoma Research Center (MRC) Mycetoma Clinic, in the surgical theatre at Soba University Hospital during the amputation procedure and during postoperative follow-up.

Semistructured in-depth, open-ended interviews were conducted. The questions covered perceptions and experiences of mycetoma, medical care, treatment accessibility, poverty and stigma. Interviews were conducted in Arabic and translated into English. All the participant observations and the in-depth interviews were audio and video recorded. The visual and audio recordings were screened several times, translated into English, transcribed, coded into meaning units, divided into categories and subcategories and analysed for structures, meaning and context. The findings and analysis were discussed.

## Patients’ stories

### El Safi said

**Figure 1.**
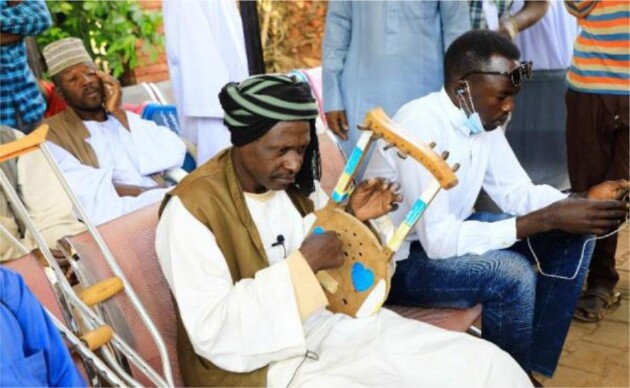
El Safi singing to the patients at the MRC.

‘My misery with mycetoma started several years back, during my traditional wedding ceremony preparation, when my sister noted a small painless mass between my toes and she asked me to consult a doctor. Because it was not painful, I ignored it and carried on with other pressing life needs, as I am a father of seven children. The mass gradually increased, then I was forced to visit the Mycetoma Research Center at Khartoum, but because of the long and expensive journey from my village in West Sudan to the center, roadblocks during the rainy seasons and expensive medications and investigations, I was not regular with the follow-up and treatment. IT WAS TOO LATE when I was forced to come back to the center. I started the medications again, which became free of charge at the center, but my movement with the wheelchair became difficult to get them. Then the COVID-19 pandemic led to movement restrictions, roadblocks and medical and health centers slowing down, so eventual amputation was the only option available for me. Thanks to God, with the support of many people and the Mycetoma Friends Association (http://mycetoma.edu.sd/mpfa/), I overcame my depression and melancholy, had a leg prosthesis and started a new job to support my family. I am coming every Monday to the center to sing to the patients and advise them on the importance of early presentation for medical care and regular follow-up’ (Figure 1).

### Albagir's brother said

**Figure 2.**
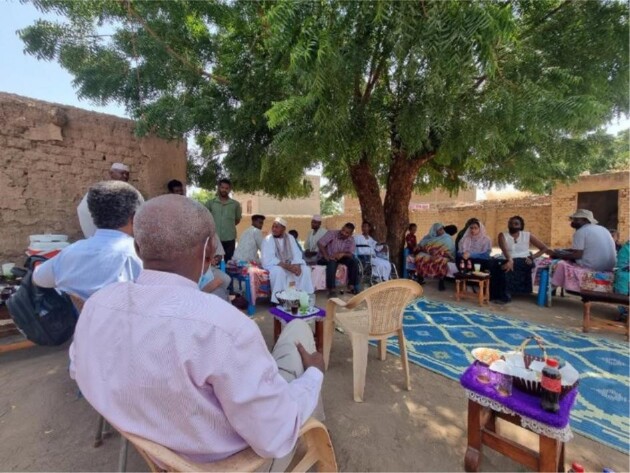
Focus group discussion with Albagir's family.

‘Albagir is a 35-year-old male from El Rawashad village at El Gaziera State. Since birth he has been a nice and pleasant physical and mental disabled patient. He is a quiet and happy person loved by the village people. He developed left foot mycetoma 20 y, and 5 y later he developed right foot mycetoma. His journey with illness was miserable and difficult. He cannot express his feelings, but he usually stops eating and movement, where his family knows there is a serious condition. His family is of low socio-economic status. It was difficult for them to take him to MRC or other medical centers for treatment. The medical doctor at his village refused to see him or to do the daily foot dressing due to the fear of contracting mycetoma. His mother, who is an elderly lady, used to do the dressing for him on a daily basis. His brother, a teacher, terminated his work contract in Oman to be with him at the village to take care of him. He was not on regular medical follow-up due to the prohibitive medication, travel expenses and recently the socio-economic, medical and security impacts of the COVID-19 pandemic on the country. He underwent below-knee amputation of the left foot and still suffering from the right foot mycetoma. His brother also had left foot mycetoma, which affected his education and eventually he had a right big toe amputation. Currently there are 69 suspected mycetoma patients in the village, 36 underwent surgical excision, and 10 patients had amputations during the COVID-19 pandemic (Figure 2).

These visual ethnographic films document the stories of two mycetoma patients and their families journeys with the disease and their unfortunate and depressing experiences and then the COVID-19 pandemic effects until the patients’ legs were amputated.^4,5^ The films then captured their recovery and a postoperative period as disabled persons with an amputation, waiting for a prosthesis that would allow them to start a new life. Despite his obvious sadness, low spirits and depression, El Safi regularly visits the MRC, mixing with and singing for the patients to improve their morale, encouraging them to continue their treatment and follow-up to avoid having the same sad outcome as he had. The film also documented the suffering experienced by Albagir and his family from the mycetoma in both feet, the increasing number of patients in their village and villages nearby and the family's willingness to start a campaign in the village to raise awareness in collaboration with the MRC. In the films produced, the patients, families and mycetoma experts presented excellent educational information on the disease.

The visual ethnographic data collected included the patients’ cultural and social contexts and interactions with healthcare providers, other patients and family and community members. This rich and enlightening data could play a crucial role in enabling a greater understanding of the daily experiences of mycetoma patients. A better appreciation of the emotions and suffering of patients could help the treating team to improve the care and socio-economic support they provide.

This visual ethnographic documentary is an innovative tool for patients, healthcare providers and community activists to articulate their feelings about and experience of mycetoma. It gives an urgently needed voice and face to the people suffering from mycetoma that will improve advocacy about and awareness of this disease, which often remains underrepresented in public discourse.

In conclusion, the documentary films produced can inform and educate various audiences and stakeholders such as policymakers, treating doctors, health workers, patients, researchers, community members and activists in the mycetoma patient community, helping them to better understand the difficult situations mycetoma patients face. The films can also be used to sensitize the international community about mycetoma and the medical and socio-economic impact of neglected tropical diseases in general.

## Authors’ contributions

AHF, LO, AHM, ESA, SMB, LO and TK designed the study protocol and film scenarios. AHF, LO, ESA, TEE, LO and TK collected the data. LO and TK filmed, edited and directed the ethnographic film. AHF, LO, SMB, MK, LO and TK drafted and revised the manuscript. All authors read and approved the final manuscript.

## Data Availability

The data are available here and online.
